# Prospective Evaluation of the BD MAX StaphSR Assay for the Screening of Methicillin-Susceptible and -Resistant *Staphylococcus aureus* from Nasal Swabs Taken in Intensive Care Unit Patients

**DOI:** 10.3390/ijms241813881

**Published:** 2023-09-09

**Authors:** Martin Fayolle, Amélie Epercieux, Cyrille H. Haddar, Sylvie Pillet, Philippe Berthelot, Bruno Pozzetto, Anne Carricajo, Florence Grattard, Paul O. Verhoeven

**Affiliations:** 1 CIRI, Centre International de Recherche en Infectiologie, GIMAP Team, Inserm, U1111, CNRS, UMR5308, ENS Lyon, Claude Bernard Lyon 1 University, 69007 Lyon, France; 2 Faculty of Medicine, Jean Monnet St-Etienne University, 42023 St-Etienne, France; 3 Department of Infectious Agents and Hygiene, University Hospital of St-Etienne, 42055 St-Etienne, France; 4 Infection Control Unit, Department of Infectious Diseases, University Hospital of St-Etienne, 42055 St-Etienne, France

**Keywords:** *Staphylococcus aureus*, methicillin resistance, MRSA, BD MAX StaphSR, nasal swab, nasal carrier, molecular techniques

## Abstract

Screening patients for *S. aureus* nasal carriage has proved effective in preventing cross-contamination and endogenous infection with this bacterium. The aim of this study was to assess the performance of the BD MAX StaphSR assay with liquid Amies elution swabs, taken during routine care of intensive care unit patients. Direct and pre-enriched cultures were used as reference methods to screen for *S. aureus* and methicillin-resistant *S. aureus* (MRSA). Discrepant results between the BD MAX StaphSR assay and cultures were resolved by using the Xpert SA Nasal Complete assay. A total of 607 nasal swabs taken from 409 patients were included in this study. Compared to culture methods, the sensitivity and specificity of the BD MAX StaphSR assay were 92.5% and 91.7% for *S. aureus* screening, and 94.7% and 98.3% for MRSA screening, respectively. In 52 (8.6%) specimens, there was a discrepancy between the results of cultures and the BD MAX StaphSR assay, including 13 (25%) where the results of the BD MAX StaphSR assay were confirmed by the Xpert SA Nasal Complete test. This prospective study showed that the BD MAX StaphSR assay is reliable for *S. aureus* and MRSA detection from nasal samples taken with liquid Amies elution swabs.

## 1. Introduction

*Staphylococcus aureus* infections are a global public health concern as this species is the leading cause of bacterial-related mortality with more than 1 million deaths per year. In France, *S. aureus* is also the leading bacterial cause of death in 2019, surpassing Enterobacteriaceae [1]. *S. aureus* is a common human commensal species colonizing around 24% of the general population [2]. The *vestibulum nasi* is recognized as the main reservoir of *S. aureus* in humans, but the entire nasal cavity is also frequently colonized [3]. *S. aureus* nasal carriers have an increased risk of endogenous infection with the *S. aureus* strain they carry. In most cases, the strain of colonization and the strain identified during the infection are genetically related [4,5]. Screening and decolonization strategies are effective in preventing *S. aureus* surgical site infections and methicillin-resistant *S. aureus* (MRSA) transmission [6,7].

Screening based on culture methods including chromogenic agar plates is useful for the detection of *S. aureus* and MRSA, but it takes several days and has lower sensitivity than Nucleic Acid Amplification Tests (NAAT) [8]. Third-generation NAAT, including the BD MAX StaphSR assay, detect *S. aureus* by amplifying a species-specific gene (e.g., the nuclease (*nuc*) gene or the staphylococcal protein A (*spa*) gene. These assays can distinguish methicillin-susceptible *S. aureus* (MSSA) from MRSA given the presence of both the methicillin-resistant gene (*mecA/C*) and the Staphylococcal Cassette Chromosome *mec* (SCC*mec*) embedded into the attB site of the *S. aureus* chromosome located at the 3′ end of *orfX*. Several studies have highlighted the excellent performances of the fully automated BD Max StaphSR assay using positive blood cultures, wound swabs, and strains collections [9,10,11,12,13,14]. Two other studies have evaluated the performances of this assay on nasal swabs for the detection of *S. aureus* and MRSA, but these studies were conducted in the United States and never in Europe, where the rate of nasal colonization by MRSA is lower than in the United States [15,16,17].

The aim of this study was to evaluate the BD MAX StaphSR assay for the detection of both MSSA and MRSA in nasal swabs taken in French intensive care units, using direct and pre-enriched cultures as the reference method.

## 2. Results

Six hundred and seven nasal Amies liquid elution swabs were included in the study. The BD MAX StaphSR assay was performed directly from the Amies liquid by transferring 200 µL into the sample buffer tube of the BD MAX StaphSR kit. In parallel, all swabs were tested by both direct and pre-enriched cultures on chromogenic agar plates to detect *S. aureus* and MRSA. Bacterial identification was confirmed by Matrix-Assisted Laser Desorption/Ionization–Time-Of-Flight Mass Spectrometry (MALDI–TOF MS). Susceptibility tests were performed by in-house PCR (*mecA* and *mecC* genes) and by cefoxitin disk diffusion test. Xpert SA Nasal Complete assays were used to analyze the discrepant results between the BD MAX StaphSR assays and culture methods. The results of culture methods combined with Xpert SA Nasal Complete assays permitted to define gold standard status for each sample.

### 2.1. Samples and Culture Methods

Based on the pre-enriched culture method, 27.9% [95% Confidence Interval (CI) 23.0–33.5%] of patients were identified as *S. aureus* nasal carriers (*n* = 114/409), among whom 9.6% [95% CI 4.8–17.3%] were MRSA carriers (*n* = 11/114), corresponding to a prevalence of 2.7% [95% CI 1.3–4.8%] (*n* = 11/409). The proportion of nasal swabs yielding *S. aureus* was 26.2% [95% CI 22.7–29.9%] (*n* = 159/607) including 11.9% [95% CI 7.2–18.7%] (*n* = 19/159) of MRSA by using the pre-enriched culture method. Pre-enriched culture enabled to recover *S. aureus* in 14 additional samples, including one sample yielding MRSA which corresponded to an increase of sensitivity of 9.7% and 5.6% for the detection of *S. aureus* and MRSA, respectively.

### 2.2. Evaluation of BD MAX StaphSR Assay

The performances of the BD MAX StaphSR assay for the detection of *S. aureus* and MRSA in nasal swabs are depicted in Table 1 and Table 2. The rate of inhibition was 0.7% [95% CI 0.2–1.7%] at the first attempt, and all samples had a valid result after retesting.

We first investigated the sensibility and specificity of the assay for *S. aureus* detection regardless of methicillin susceptibility. The BD MAX StaphSR assay detected *S. aureus* in 44 samples detected negative by the direct culture method (Table 1 and Table A1). Of these samples, the pre-enriched culture recovered *S. aureus* in 7 samples, and 13 other samples were tested positive for *S. aureus* with the Xpert SA Nasal Complete assay. Conversely, pre-enriched culture recovered *S. aureus* in 12 samples that were not detected positive by the BD MAX StaphSR assay (Table A2). The Xpert SA Nasal Complete assay detected MSSA in 5 of these samples. In few cases, the pre-enriched culture method enabled to recover *S. aureus* in NAAT negative samples. In contrast, the BD MAX StaphSR assay detected *S. aureus* in 24 samples that were negative by culture and Xpert SA Nasal Complete assay (Table 1). The mean cycle threshold (CT) value (± SD) of *nuc* target for these samples was 33.1 ± 3.4, including five samples with CT > 37, which indicate a low *S. aureus* load.

Next, we examined the performance of the BD MAX StaphSR assay for the detection of MRSA (Table 2 and Table A1). All samples recovering MRSA by direct culture were detected positive for MRSA by the BD MAX StaphSR assay. One further sample was tested positive for MRSA by pre-enriched culture, but this sample was not detected as positive by either the BD MAX StaphSR nor the Xpert SA Nasal Complete assays (Table A2). The strain isolated from this sample was correctly identified as MRSA by the BD MAX StaphSR assay. On the other hand, seven culture-negative samples were found to be positive for MRSA by the BD MAX StaphSR assay. The targets used to define the presence of MRSA (i.e., *spa*, *mecA*, SCC*mec*) were amplified by the Xpert SA Nasal Complete assay in two samples, but only one sample was interpreted as positive by the GeneXpert Dx System software (version 2.1). In five of these samples, only the SCC*mec-orfX* right-extremity junction (MREJ) and *mecA/C* targets were detected on the BD MAX system. The difference between the CT values of both targets exceeded 2 CT for four of these samples. For the three remaining discrepant cases, the presence of MSSA was detected by culture and Xpert SA Nasal Complete assay. To further investigate these three cases, the BD MAX StaphSR assay was performed directly from the three *S. aureus* strains recovered by culture. In both cases, the *S. aureus* species-specific and the MREJ targets, but not the *mecA/C* target, were detected directly from the bacterial strains, which suggests that these strains were MRSA *mecA* dropout genotypes. These nasal swabs were suspected to contain a mixture of MRSA *mecA* dropout and methicillin-resistant coagulase-negative staphylococci (MR CoNS). Based on the sample status defined by the gold standard of this study, the sensitivity and specificity of BD MAX StaphSR assay for MRSA detection were 95.0% [95% CI 75.1–99.9%] and 98.5% [95% CI 97.1–99.3%], respectively (Table 2). 

## 3. Discussion

*S. aureus* is still a leading cause of bacterial-related mortality and a major agent of nosocomial infections. This bacterium has developed important drug-resistance mechanisms over the decade [1,18]. Bundled infection control programs involving screening for *S. aureus* and MRSA colonization by NAATs (at least one nasal swab plus one perineal or one throat swab) combined with decolonization have shown significant efficiency in reducing the spread and surgical site infections [1,6,7,19]. These screening tests need to be repeated over time, as decolonization of the nose and other colonization sites is successful but short-lived [19,20]. The use of effective screening methods is essential. In 2018, the World Health Organization recommended perioperative decolonization for all patients undergoing cardiothoracic and orthopaedic surgery (strong recommendations) or other types of surgery (conditional recommendations) with known nasal carriage of *S. aureus* [21]. The implementation of a screening and decolonization strategy and an antimicrobial stewardship program [22] is crucial for reducing *S. aureus* infections, antibiotic consumption, length of hospital stay, and readmissions.

Our prospective study has shown that the BD MAX StaphSR assay is reliable for detecting both MSSA and MRSA colonization from nasal swabs in intensive care patients. Several studies were carried out to evaluate the BD MAX StaphSR assay for the detection of *S. aureus* and MRSA directly from samples for different indications. From positive blood cultures, the sensitivity and specificity were found to range from 99.4% to 100% and 99.5% to 100% for *S. aureus* detection, and from 97.9% to 100% and 98.1% to 100% for MRSA detection, respectively [9,10,11]. It is worth noting that Dalpke et al. identified six *S. aureus* culture-negative bottles with a false-positive BD MAX StaphSR test result that were considered to be a bench-level contamination, thus overestimating our calculated range of specificity. Overall, the performance from positive blood cultures is better than in our study, which is probably explained by the high *S. aureus* load in positive blood cultures. Another study carried out on 250 wound swabs taken from US patients showed a sensitivity of 100% and 98.2% and a specificity of 95.2% and 99.5% for the detection of *S. aureus* and MRSA, respectively, which is slightly higher than the results observed in our cohort [12]. Finally, only two published studies have evaluated the BD MAX StaphSR assay for the screening of nasal carriers. These two studies reported a sensitivity of 96.4% and 93.6% for *S. aureus* and MRSA detection, respectively, and a specificity of 93.1% and 97.7% for *S. aureus* and MRSA detection, respectively [15,16]. As both studies were carried out in North America, the prevalence of MRSA nasal carriage was high due to the widespread of the community-acquired methicillin-resistant USA300 clone, and most MRSA isolates may belong to the USA300 lineage [15,16,17]. In contrast, our study is the first to be carried out in Europe to evaluate the performance of the BD MAX StaphSR assay for MRSA and MSSA nasal carriage screening. Although the spreading of MRSA clones is strikingly different in Europe and North America, the BD MAX StaphSR assay showed excellent sensitivity and specificity to detect *S. aureus* and MRSA nasal carriers in our intensive care units. Moreover, the prevalence of *S. aureus* and MRSA nasal carriage was consistent with those observed in similar studies performed in Europe [2].

Although the results of our study were excellent, there are some limitations. As this study was monocentric in a cohort of intensive care patients, local epidemiology may have influenced the results. In addition, the SA Xpert Nasal Complete assay was performed only on specimens that gave discrepant results, which may lead to a bias in the definition of the gold standard status of the specimens. 

Interestingly, we observed a few cases with controversial results between the techniques used in our study. Firstly, the BD MAX StaphSR assay detected MRSA in five culture-negative samples, while the *nuc* target was not detected. In fact, the BD Max StaphSR assay identifies MRSA when both MREJ and *mecA*/*C* are detected together, regardless of the result of the *nuc* target, with the aim of not missing MRSA strains [10]. Ellem et al. and Van Leeuwen et al. reported strains having a divergent *nuc* gene which did not amplify with *nuc* primers but was *femA*-positive [10,23]. Such strains have been recognized in the past, and the inability of this assay to amplify *nuc* has also been previously reported in MRSA strains [23], but this phenomenon seems to occur with a very low frequency worldwide. However, as the strains were not obtained by culture, it was not possible to decipher these false-positive MRSA cases. Secondly, of the three samples we suspected of containing a mixture of *mecA* dropout *S. aureus* and MR CoNS, one sample showed dissociated CT values between SCC*mec* and *mecA/C* targets, which suggests the presence of MR CoNS in this sample. Unfortunately, in the two remaining cases, the CT values of the targets were too close to suspect a mixture. Indeed, third-generation PCR assays targeting the *SCCmec-orfX* right-extremity junction and the *mecA* gene were found to reduce the occurrence of false-positive results due to *mecA* dropout, but they do not eliminate it in the case of patients colonized by both *S. aureus*-*mecA* dropout and the CoNS-carrying *mecA* gene. In a collection of strains isolated in the US, 7.1% of MSSA isolates were compatible with the genotype of the *S. aureus* empty-cassette variant [13]. Lee et al. reported a prevalence of *mecA* dropout-*S. aureus* of 4.8% in atopic dermatitis samples from a Korean cohort [24]. Unfortunately, we did not note the presence of Coagulase-Negative Staphylococci (CoNS) in culture. The prevalence of *mecA* dropout-*S. aureus* not being neglectable, the algorithms for interpreting NAAT still need to be refined to avoid false-positive results when these isolates are mixed with MR CoNS. Lastly, one MRSA culture-positive sample was missed by both BD MAX StaphSR and Xpert Nasal Complete assays. Although sequence variations in the SCC*mec-orfX* junction previously described lead to false-negative MRSA results in other NAATs [25,26], the false-negative result observed in our study was likely due to a very low *S. aureus* load in the sample, as the strain was correctly identified as MRSA by the BD MAX StaphSR assay. 

Another study has shown that liquid Amies elution swabs are suitable to be used with the BD Max StaphSR assay [15]. The excellent performance and the low level of inhibition observed in our cohort confirm that liquid Amies elution swabs can be used with the BD Max StaphSR assay in clinical practice. 

## 4. Materials and Methods

### 4.1. Patients and Samples

A total of 607 nasal swabs (eSwab ref. 480 CE and 484 CE, Copan, Brescia, Italy) were included in this prospective study. Samples were collected consecutively without selection criteria during routine care from 409 intensive care patients, including children and adults, at the University Hospital of Saint-Etienne, France. All samples were tested by both the BD MAX StaphSR assay, direct culture, and pre-enriched culture.

### 4.2. BD MAX StaphSR Assay

Following the manufacturer’s recommendations, swabs were vortexed briefly and a 200 µL volume of Amies liquid was added directly into the sample buffer tube provided with the kit. The BD MAX StaphSR assay has been designed to detect 3 targets including the *nuc* gene, the SCC*mec-orfX* right-extremity junction (MREJ), and the *mecA/C* gene. The result of the amplification of these 3 targets was automatically interpreted by the algorithm of the BD MAX system.

### 4.3. Culture Methods, Identification, and Susceptibility Tests

All samples were tested by both direct and pre-enriched cultures for MSSA and MRSA detection. For direct culture, swabs were streaked on chromogenic agar plates dedicated to the screening of *S. aureus* (BBL CHROMagar Staph aureus, Becton Dickinson, Le Pont de Claix, France) and MRSA (BBL CHROMagar MRSA II, Becton Dickinson). Agar plates from direct culture were incubated at 36 °C for 48 h. For pre-enriched culture, a 6.5% NaCl broth (BBL Salt Broth, modified, Becton Dickinson) was inoculated with 100 µL of sample and incubated at 36 °C overnight. If the broth was growing, a 10 µL volume of broth was streaked onto chromogenic agar plates (BBL CHROMagar Staph aureus and BBL CHROMagar MRSA II, Becton Dickinson) and incubated at 36 °C overnight. Presumptive colonies of *S. aureus* or MRSA were identified by MALDI–TOF MS (Microflex LT, Bruker Daltonics, Bremen, Germany). All *S. aureus* isolates on MRSA chromogenic agar plates were tested for methicillin resistance by using a cefoxitin susceptibility test and an in-house PCR assay targeting the *nuc*, *mecA*, and *mecC* genes [27].

### 4.4. Definition of S. aureus Nasal Carriage Status

Samples with discrepant results between the BD MAX StaphSR assay and the culture methods were tested with the Xpert SA Nasal Complete assay (Cepheid, Maurens-Scopont, France). Samples were considered positive for *S. aureus* or MRSA if at least the cultures or the Xpert SA Nasal Complete assay detected *S. aureus* or MRSA, respectively. The results of direct and pre-enriched cultures combined to the result of the Xpert SA Nasal Complete assay were considered as the gold standard status. Statistical analyses were performed using the MedCalc Statistical Software version 22.007 (MedCalc Software Ltd., Ostend, Belgium).

## 5. Conclusions

To conclude, the BD MAX StaphSR assay is a fully automated third-generation NAAT that enables rapid and accurate detection of *S. aureus* and MRSA nasal carriers and could help to reduce both nosocomial and endogenous infections by *S. aureus*.

## Figures and Tables

**Table 1:** Performance of the BD MAX StaphSR assay for the detection of *S. aureus* according to the sample status defined by different techniques.

Reference Technique	Sample Status	BD MAX StaphSR Assay					
		*S. aureus*-Positive (*n*)	*S. aureus*-Negative (*n*)	Sensitivity [95% CI] (%)	Specificity [95% CI] (%)	NPV ^1^ [95% CI] (%)	PPV ^2^ [95% CI] (%)
Direct culture only	*S. aureus*-positive	140	5	96.6 [92.1–98.9]	90.5 [87.4–93]	98.8 [97.2–99.5]	76.1 [70.6–80.8]
	*S. aureus*-negative	44	418				
Both direct and pre-enriched cultures	*S. aureus*-positive	147	12	92.5 [87.2–96]	91.7 [88.8–94.1]	97.2 [95.2–98.3]	79.9 [74.4–84.4]
	*S. aureus*-negative	37	411				
Both direct and pre-enriched cultures combined w/Xpert PCR assay	*S. aureus*-positive	160	12	93.0 [88.1–96.3]	94.5 [91.9–96.4]	97.2 [95.2–98.3]	87.0 [81.8–90.8]
	*S. aureus*-negative	24	411				

^1^ NPV: Negative predictive value. ^2^ PPV: Positive predictive value.

**Table 2:** Performance of the BD MAX StaphSR assay for the detection of methicillin-resistant *S. aureus* (MRSA) according to the sample status defined by different techniques.

Reference Technique	Sample Status	BD MAX StaphSR Assay					
		MRSA-Positive (*n*)	MRSA-Negative (*n*)	Sensitivity [95% CI] (%)	Specificity [95% CI] (%)	NPV ^1^ [95% CI] (%)	PPV ^2^ [95% CI] (%)
Direct culture only	MRSA-positive	18	0	100 [81.5–100]	98.3 [96.9–99.2]	100 [99.4–100]	64.3 [49.3–76.9]
	MRSA-negative	10	579				
Both direct and pre-enriched cultures	MRSA-positive	18	1	94.7 [74–99.9]	98.3 [96.9–99.2]	99.8 [98.8–100]	64.3 [49.1–77.1]
	MRSA-negative	10	578				
Both direct and pre-enriched cultures combined w/Xpert PCR assay	MRSA-positive	19	1	95.0 [75.1–99.9]	98.5 [97.1–99.3]	99.8 [98.8–100]	67.9 [52.3–80.3]
	MRSA-negative	9	578				

^1^ NPV: Negative predictive value. ^2^ PPV: Positive predictive value.

## Data Availability

If additional data related to this study are required, please consult the corresponding author.

## Appendix A

**Table A1:** Diagnostic table according to the result of the reference technique.

Reference Technique	Sample Status	BD MAX StaphSR Results			
		All *n* = 607	MSSA ^1^ *n* = 156	MRSA ^2^ *n* = 28	Negative *n* = 423
Direct culture only	MSSA	127	120	2	5
	MRSA	18	0	18	0
	Negative	462	36	8	418
Both direct and pre-enriched cultures	MSSA	140	126	3	11
	MRSA	19	0	18	1
	Negative	448	30	7	411
Both direct and pre-enriched cultures combined w/Xpert PCR assay	MSSA	152	138	3	11
	MRSA	20	0	19	1
	Negative	435	18	6	411

^1^ MSSA: methicillin-susceptible *S. aureus*. ^2^ MRSA: methicillin-resistant *S. aureus*.

**Table A2:** Results of samples showing discrepant results (*n* = 52) with the BD MAX StaphSR assay, the direct and pre-enriched culture, and the Xpert SA Nasal Complete assay.

No.	Both Direct and Pre-Enriched Culture	Xpert SA Nasal Complete Assay					BD MAX StaphSR Assay				
		CT Value					CT Value				
		*spa*	*mecA*	SCC*mec*	SPC ^4^	Results	*nuc*	*mecA/C*	SCC*mec*	SPC	Results
112	MRSA ^1^	0.0	30.7	0.0	33.1	SA −	-	31.4	-	33.0	SA −
108	MSSA ^2^	24.8	0.0	30.2	33.3	MSSA	17.6	26.4	18.9	29.6	MRSA
068	MSSA	27.8	0.0	32.0	32.4	MSSA	23.7	23.5	24.5	31.0	MRSA
034	MSSA	34.7	0.0	-	0.0	MSSA	32.6	32.6	33.7	30.6	MRSA
009	MSSA	35.1	17.4	-	35.4	MSSA	-	18.8	-	30.1	SA −
067	MSSA	31.0	17.7	-	39.7	MSSA	-	20.0	-	26.4	SA −
162	MSSA	34.2	32.1	-	33.3	MSSA	-	27.9	-	-	SA −
424	MSSA	34.2	33.7	-	32.9	MSSA	-	-	-	31.9	SA −
501	MSSA	34.8	30.5	-	33.2	MSSA	-	29.1	-	30.9	SA −
002	MSSA	0.0	19.9	0.0	33.2	SA −	-	21.8	-	26.0	SA −
053	MSSA	0.0	36.6	0.0	32.5	SA −	-	34.1	-	29.2	SA −
118	MSSA	37.0	30.3	-	35.5	SA −	-	25.7	-	30.4	SA −
155	MSSA	-	-	-	31.7	SA −	-	-	-	31.3	SA −
351	MSSA	36.1	36.6	-	33.5	SA −	-	34.7	-	30.8	SA −
370	MSSA	-	28.3	-	34.4	SA −	-	27.0	-	29.6	SA −
519	SA − ^3^	31.4	31.9	33.3	33.3	MRSA	31.3	31.6	32.0	31.5	MRSA
015	SA −	33.2	31.6	-	33.1	MSSA	34.1	31.2	-	28.3	MSSA
029	SA −	31.0	32.5	-	32.9	MSSA	32.2	32.7	-	29.9	MSSA
066	SA −	33.4	37.8	-	35.9	MSSA	33.1	29.7	-	30.7	MSSA
071	SA −	33.1	27.4	-	33.1	MSSA	38.0	24.9	-	29.9	MSSA
212	SA −	32.9	31.5	-	32.8	MSSA	31.0	31.0	-	30.9	MSSA
221	SA −	31.1	25.1	-	33.4	MSSA	28.9	27.9	-	30.9	MSSA
276	SA −	33.4	23.2	-	-	MSSA	28.9	25.2	-	29.1	MSSA
421	SA −	33.3	26.5	-	32.1	MSSA	31.9	28.0	-	31.5	MSSA
451	SA −	30.0	-	-	32.5	MSSA	29.3	-	-	31.3	MSSA
495	SA −	32.9	-	-	34.7	MSSA	28.4	-	-	29.8	MSSA
509	SA −	33.4	31.2	-	34.6	MSSA	30.0	29.0	-	29.8	MSSA
598	SA −	33.4	28.7	-	32.9	MSSA	32.7	29.3	-	31.2	MSSA
317	SA −	35.4	34.0	37.9	33.2	SA −	33.4	33.5	32.1	31.0	MRSA
007	SA −	0.0	0.0	0.0	35.3	SA −	-	37.9	23.9	26.9	MRSA
023	SA −	0.0	30.9	0.0	32.8	SA −	-	27.2	31.1	29.0	MRSA
144	SA −	0.0	25.1	0.0	31.7	SA −	-	27.3	19.1	29.5	MRSA
491	SA −	-	36.2	39.1	34.4	SA −	-	34.3	31.4	30.3	MRSA
561	SA −	-	33.2	-	32.0	SA −	-	34.6	33.6	31.2	MRSA
057	SA −	-	-	-	32.9	SA −	37.6	-	-	29.9	MSSA
214	SA −	-	-	-	33.5	SA −	32.0	-	-	29.9	MSSA
293	SA −	-	28.7	-	31.7	SA −	32.5	30.8	-	30.3	MSSA
328	SA −	-	37.3	-	33.3	SA −	35.1	-	-	31.4	MSSA
341	SA −	35.4	22.7	-	31.6	SA −	38.0	25.7	-	30.2	MSSA
353	SA −	-	26.6	-	34.5	SA −	33.2	28.0	-	31.1	MSSA
357	SA −	-	26.4	-	34.3	SA −	37.1	37.6	-	31.5	MSSA
420	SA −	-	-	-	34.4	SA −	32.1	-	-	31.0	MSSA
425	SA −	-	24.2	-	34.2	SA −	28.9	25.5	-	30.0	MSSA
430	SA −	-	-	-	33.3	SA −	26.3	-	-	31.5	MSSA
435	SA −	-	30.4	-	33.3	SA −	30.8	31.5	-	30.8	MSSA
442	SA −	-	26.1	-	32.0	SA −	32.7	28.3	-	30.2	MSSA
449	SA −	36.7	33.2	-	33.7	SA −	36.4	31.7	-	29.2	MSSA
470	SA −	-	34.2	-	33.2	SA −	35.4	-	-	31.8	MSSA
504	SA −	35.1	24.0	-	-	SA −	36.4	25.9	-	30.9	MSSA
546	SA −	-	33.0	-	35.1	SA −	33.8	31.8	-	29.8	MSSA
584	SA −	38.0	34.7	-	33.1	SA −	30.5	29.3	-	29.6	MSSA
590	SA −	35.9	-	-	33.2	SA −	27.3	-	-	27.6	MSSA

^1^ MRSA: methicillin-resistant *S. aureus*. ^2^ MSSA: methicillin-susceptible *S. aureus*. ^3^ SA −: Detection of *S. aureus*-negative. ^4^ SPC: Sample Processing Control.
